# Alcohol facilitates detection of concealed identity information

**DOI:** 10.1038/s41598-018-25811-z

**Published:** 2018-05-18

**Authors:** Kristina Suchotzki, Matthias Gamer

**Affiliations:** Department of Psychology, University of Würzburg, Marcusstr. 9-11, 97080 Würzburg, Germany

## Abstract

The Concealed Information Test (CIT) is a well-validated means to detect whether someone possesses certain (e.g., crime-relevant) information. The current study investigated whether alcohol intoxication during CIT administration influences reaction time (RT) CIT-effects. Two opposing predictions can be made. First, by decreasing attention to critical information, alcohol intoxication could diminish CIT-effects. Second, by hampering the inhibition of truthful responses, alcohol intoxication could increase CIT-effects. A correlational field design was employed. Participants (*n* = 42) were recruited and tested at a bar, where alcohol consumption was voluntary and incidental. Participants completed a CIT, in which they were instructed to hide knowledge of their true identity. BAC was estimated via breath alcohol ratio. Results revealed that higher BAC levels were correlated with higher CIT-effects. Our results demonstrate that robust CIT effects can be obtained even when testing conditions differ from typical laboratory settings and strengthen the idea that response inhibition contributes to the RT-CIT effect.

## Introduction

A common situation in police interrogations is that a crime suspect hides critical information from the investigators. This information can be directly related to a committed crime (e.g., the transport vehicle used to flee from a robbery), information about a planned crime (e.g., the place of a planned terroristic attack) or identity information (e.g., the identity of the suspect or accomplices). In those situations, the Concealed Information Test (CIT)^1^ provides a well-validated means to probe for such knowledge^2^. In cases in which the investigators know the critical piece of information (e.g., the murder weapon), a suspect is presented with this probe (e.g., a knife) amongst a number of well-matched neutral stimuli (i.e., other potential weapons). More pronounced responding to the probe compared to the neutral stimuli provides the investigators with an indication that the suspect possesses incriminating knowledge. In cases in which the investigators do not know the critical information (e.g., the place of a planned attack or the identity of accomplices), several potential pieces of information (e.g., places or names) can be probed and stronger responding to one of the alternatives can guide further investigations (the searching CIT; see e.g., ref.^3^). Both versions of the CIT are used on a regular basis in police investigations in Japan, with about 5000 tests being conducted per year^4^.

Both in applied settings as well as in research, the CIT has traditionally been used with autonomic response measures^1^. A recent meta-analysis, however, also revealed a large reaction time (RT) CIT-effect (*d* = 1.22)^5^, with longer reaction times for probes compared to neutral stimuli. Yet, CIT meta-analyses also revealed considerable variation in the size of CIT effects between studies, indicating a need to identify factors that may moderate CIT-effects^5,6^.

Although not included in previous meta-analyses due to a lack of available research, one potential moderator of the CIT-effect may be alcohol intoxication. This is interesting from both an applied perspective, as alcohol has been proposed as a potential countermeasure that suspects may use to counteract CIT effects^7,8^, as well as from a theoretical perspective. Based on theories, there are two directions in which alcohol intoxication during the test could alter CIT-effects. Traditionally, enhanced responding to critical information has been explained with orienting and attentional mechanisms^9^. Interestingly in this context, previous studies investigating the influence of alcohol on attention have shown that alcohol decreases the percentage of correct identifications of relevant stimuli^10^, especially when a second task has to be completed at the same time^11^. Alcohol myopia theory^12^ explains this by proposing that alcohol reduces the overall attentional capacity, and thereby leads people to focus all resources on a primary task. In RT-CITs, suspects are usually required to identify a number of crime-irrelevant target stimuli that are learned before the testing procedure and acknowledge their recognition with a specific “yes”-button. Those target stimuli are embedded in a series of neutral stimuli and crime-relevant probes. Based on alcohol myopia theory, alcohol may lead participant to focus on correctly solving the seemingly primary task that is a rapid and correct identification of target stimuli, which may in turn limit their attention towards crime-related probes. However, recent research also suggests that response inhibition may contribute to the enhanced responses to probes compared to neutral information^13^. As explained above, in RT-CITs, suspects are usually required to admit knowledge of target stimuli, while actively denying knowledge of the crime-relevant probes. Based on research on lying, it has been proposed that such a “no”-response to actually recognized information requires the inhibition of the truthful “yes”-response^14–17^, thereby increasing error rates and slowing RTs. As it is well-known from the response inhibition literature that acute alcohol intoxication hampers response inhibition^18–20^ (for a review see also ref.^21^), it may also hamper deceitful responding in the CIT. To sum up, alcohol myopia theory may predict smaller CIT-effects during alcohol intoxication, due to a focus on target identification and a limited attention towards probes. In contrast, response inhibition theory would predict larger CIT-effects during alcohol intoxication, due to a more difficult deceitful responding to probes.

The only two studies that investigated the effect of alcohol intoxication on the CIT used autonomic response measures^7,22^. Only Bradley and Ainsworth (1984)^7^ manipulated alcohol intoxication during the actual CIT, yet did not find any effect of the manipulation. The authors only found significant effects for alcohol intoxication during the preceding mock crime during which the probe items were encoded. This effect was most likely driven by the effects of alcohol on memory, although the data are not clear as this effect could not be replicated in a later study by O’Toole, *et al*^22^.

In the current study, we investigated the effect of alcohol intoxication during an RT-CIT. To control for memory effects, we used participants’ identity stimuli as probes in the CIT, as those stimuli are likely to be very deeply encoded. To study effects in a natural drinking environment, the experiment took place in a bar and night club. Note that the current study is similar to an earlier study conducted at a science festival, which found no effects of acute alcohol intoxication on the reaction time cost of lying^13^. In this study, however, the authors employed a deception paradigm in which truth telling and lying was contrasted and not an RT-CIT, which combines the effects of recognition and deception. Furthermore, the alcohol intoxication in this earlier study was relatively low, raising the question to what extend higher intoxication levels may affect deceitful responding.

## Method

### Participants

In total, 48 visitors of the student party at the Zauberberg (a combined bar and night club in Würzburg, Germany) volunteered to participate in the study. The study conformed to the principles expressed in the Declaration of Helsinki and was approved by the Ethics Committee of the Institute of Psychology of the University of Würzburg. All participants provided written informed consent. One participant did not complete the entire experiment. Data of four participants were excluded from data-analyses because of reported or suspected drug use. Data of one participant were excluded because of high error rates in the CIT (exceeding the group mean error rate per item plus 2.5 *SDs*). The mean age of the remaining 42 participants was 23.26 years (*SD* = 4.52 years; 13 female, 31 male).

### Procedure

Testing took place on four evenings from 11.00 PM to approximately 2.00 AM. The study was advertised as opportunity to show who had the potential to be a good secret agent (the “new James Bond”) and to that means successfully take on a new “cover” identity and hide one’s true identity. Everyone interested in the study could participate. Participants were not selected on the basis of their alcohol consumption and were not encouraged to drink alcohol.

Participants first filled out a questionnaire assessing demographic variables (sex, age and education), and the five identity stimuli that served as probe items. They then completed a short pre-test procedure and the CIT (see below). After completion of the test, feelings of tension, boredom, tiredness, intoxication, perceived test difficulty (1–5 Likert scales), drinking behavior (number of alcoholic consumptions) and drug use on that evening (yes/no) were assessed. Testing took place on two laptops placed on a standing table in the bar of the night club, with two participants being simultaneously tested. Participants wore sound dampening headphones during the experiment and were not allowed to drink to ensure a minimum of 15 minutes (i.e., the approximate duration of the experiment) between the last alcoholic drink and the alcohol test. At the end of the experiment, participants were asked to drink a sip of water and blood alcohol concentration (BAC) was estimated with the Dräger Alcotest 3000. Participants were instructed to exhale into the Alcotest device until they heard a clicking sound. The Dräger Alcotest 3000 then immediately converted the breath alcohol ratio into BAC in %. Finally, participants were told their BAC values. If participants were severely intoxicated, they were warned about the consequences of severe alcohol intake and advised to stop drinking. Participants were thoroughly debriefed about the purpose and the background of the experiment.

### Concealed Information Test

The pre-test procedure and the CIT were presented with Inquisit 4. During the pre-test procedure, participants were presented with five stimuli belonging to their new “cover” identity. These stimuli served as target stimuli during the test and were “Name: Sophie/Peter” (depending on the sex of the participant), “Birthday: 8. May”, “Birth town: Lübeck”, “Name of mother: Ulrike”, “Name of father: Erich”. Participants were told to remember those stimuli thoroughly, so that during the CIT, they could successfully pretend to be this person. A screen with all five stimuli was presented twice for 30 seconds. After each disappearance of the screen, participants had to type in all five stimuli via the keyboard. In the CIT, participants were then told that the experimenters were aiming to “uncover” their real identity. Participants were instructed that they would see a number of identity stimuli and that they had to indicate whether they recognized them or not. Recognition should only be acknowledged for the previously learned target stimuli (by pressing the “yes” key) and be denied for all other identity stimuli, including the stimuli referring to their actual identity (by pressing the “no” key). The “a” and the “l” key of a standard QWERTZ keyboard were used, with the assignment of “yes” and “no” responses being counterbalanced between participants.

In total, 30 different stimuli (the five aforementioned target stimuli, five probe stimuli consisting of the actual identity stimuli of the participants and 20 neutral stimuli [i.e., other names, dates and German towns]) were each presented six times in completely randomized order (180 trials in total). Reminder labels for “yes” and “no” responses appeared on the left and right lower part of the screen. Participants were instructed to respond as fast and correctly as possible. If participants did not respond after 2500 ms, the words ‘Too slow’ were presented centrally on the screen. Those trials were not repeated later on. The inter trial interval was set to vary randomly between 500, 600, 700, 800, 900 and 1000 ms. After 90 trials, participants could take a self-paced break.

## Results

Data were analyzed with R software and raw data as well as the analysis script can be accessed on https://osf.io/6fa2h/. There were 3 participants for which one probe was identical with one target (e.g., name of the father) and those items were excluded. Also, trials exceeding the response deadline were excluded (0.65%). All further analysis steps can be found below. For t-tests, the standardized mean difference *d* was calculated as measure of effect size, with 0.20, 0.50 and 0.80 indicating ‘small’, ‘moderate’ and ‘large’ effects^23^. For paired t-tests, *d* was corrected for the intercorrelation of values (Cohen’s *d* for paired data)^24^. The distribution of BAC had a slight overrepresentation of BAC = 0.00% (*n* = 10), yet measures of skewness and kurtosis did not reveal a significant deviation from normality (z_skewness_ = 0.23, *p* = 0.629; z_kurtosis_ = -0.75, *p* = 0.773). BACs ranged between 0.00% and 0.19%, with an average BAC of 0.06% (*SD* = 0.05; *Mdn* = 0.07).

Means and standard deviations of all other assessed variables can be found in Tables 1 and 2. Paired sample t-tests confirmed that probes resulted in a higher error percentage than neutral stimuli, *t*(41) = 3.60, *p* = 0.001, *d* = 0.56. After removal of error trials and RT outliers (3.06%; RTs > 2.5 *SD*s from the mean per subject and item type), paired sample t-tests confirmed that probes resulted in longer RTs compared to neutral stimuli, *t*(41) = 9.69, *p* < 0.001, *d* = 1.50. Note that not removing RT outliers did not change this pattern of results.

**Table 1:** Mean error rate and reaction time for neutral stimuli, probes and targets.

	Neutral stimuli	Probes	Targets
Error rate (in %)	1.45 (3.33)	4.26 (5.51)	16.85 (10.56)
Reaction times (in ms)	601.81 (107.38)	700.66 (140.18)	746.60 (112.04)

*Note*. Standard deviations are given in brackets.

**Table 2:** Means and Standard deviations of all assessed variables and Correlations (*r*) with BAC.

Measure	*M*	*SD*	*r*(42) with BAC
BAC	0.06	0.05	—
Intoxication	2.26	0.99	0.67***
No. consumptions	4.91	3.40	0.75***
Tension	1.86	1.07	0.11
Anxiety	3.08	0.82	0.06
Tiredness	2.24	0.91	−0.01
Difficulty	2.12	0.80	−0.01
ER CIT-effect	2.80	5.04	0.11
RT CIT-effect	98.85	66.10	0.39*

*Note*. BAC = Blood alcohol concentration, No. consumptions = Number of alcohol consumptions on that day, ER = Error rate in %, RT = Response time in ms, CIT-effect = Differences between probes and neutral items. *p*-values reported two-tailed. *p < 0.05. ***p < 0.001.

As manipulation check, we computed the correlation (*r*) between BAC and the feeling of intoxication and between BAC and the number of alcohol consumptions. Note that *r* also serves as effect size, with 0.10, 0.30 and 0.50 indicating ‘small’, ‘moderate’ and ‘large’ effects. As can be seen in Table 2, higher levels of BAC were related to a higher feeling of intoxication and a larger number of reported alcoholic consumptions. We also computed the correlations between BAC and the feelings of tension, anxiety, tiredness, and perceived test difficulty. As can be seen in Table 2, none of those analyses revealed significant effects.

**Figure 1:**
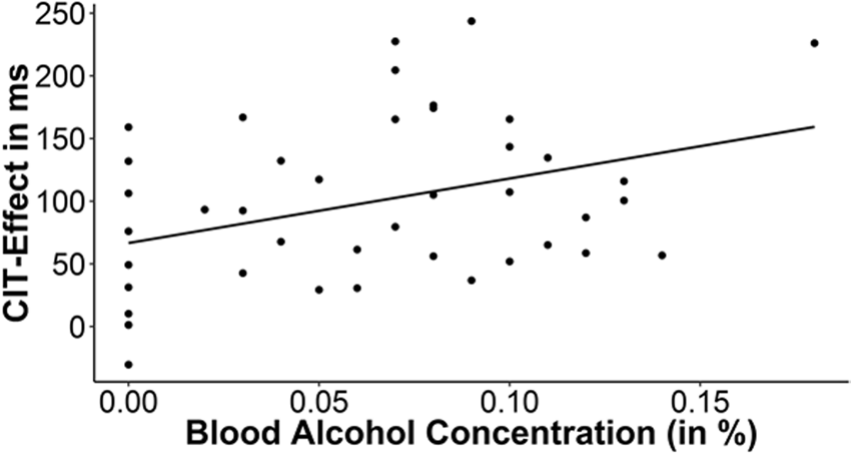
Scatter plot displaying the mean blood alcohol concentration in relation to the mean CIT-effects. Note that the upper right value with a BAC of 0.19% could be considered an outlying value. Diminishing the influence of this value by using a non-parametric correlation coefficient (*Spearman’s rho* = 0.33, *p* = 0.031) or computing a robust regression (with iterated re-weighted least squares (IRLS) and Huber weights, *t*(40) = 2.338, *p* = 0.024) still reveals a significant relationship.

To investigate the link between BAC and the CIT effect, CIT effects were computed by subtracting error percentages for neutral stimuli from probes (i.e., ER CIT-effect) and RTs for neutral stimuli from probes (RT CIT-effect). As can be seen in Table 2 and Fig. 1, higher levels of BAC were related to higher RT CIT-effects. Using a median split to divide participants into a group with low (BAC ≤ 0.07) and high (BAC > 0.07) BAC levels confirms those results. RT CIT-effects were larger in the group with high BAC levels (*M* = 123 ms, *SD* = 63 ms) compared to the group with lower BAC levels (*M* = 75 ms, *SD* = 62 ms), *t*(40) = 2.49, *p* = 0.017, *d* = 0.77.

To discriminate between effects of acute alcohol consumption and possible confounding variables, we also computed the partial correlations between BAC and the RT CIT-effect, controlling for the feelings of tension, anxiety, tiredness, and perceived test difficulty. With *r*(42) = 0.37, *r*(42) = 0.44, *r*(42) = 0.40, *r*(42) = 0.41, all correlations remained significant (all *p*’s < 0.05).

Based on the suggestion of a reviewer, we also looked at the results of the target trials, as correlations of BAC with performance on target trials may be indicative of a more general effect of alcohol on working memory capacity and action selection. As usual in RT-CITs, paired sample t-tests confirmed that targets resulted in higher error rates compared to neutral stimuli, *t*(41) = 9.71, *p* < 0.001, *d* = 1.50, and compared to probe stimuli, *t*(41) = 7.91, *p* < 0.001, *d* = 1.22. Targets also resulted in longer RTs compared to neutral stimuli, *t*(41) = 13.23, *p* < 0.001, *d* = 2.04, and compared to probe stimuli, *t*(41) = 3.56, *p* < 0.001, *d* = 0.55. There were, however, no significant correlations between differences of target and neutral stimuli with BAC levels, neither for error rates, *r*(42) = 0.30, *p* = 0.057, nor for RTs, *r*(42) = 0.09, *p* = 0.572.

## Discussion

The current study undertook a first step to investigate the influence of alcohol on the detection of concealed information. To that means, we used a correlational field design, in which participants were recruited and tested in a night club in which alcohol consumption was voluntary.

The first notable result of our study is the observation of large RT CIT-effects obtained in a high noise environment with part of the participants even being intoxicated (*d* = 1.50). Other research has already shown that RT-CITs can be successfully administered via the internet^25^ and our results strengthen the conclusion that robust CIT effects can be obtained even when testing conditions differ from typical laboratory settings and when participants may not be fully concentrated. The second notable result is the correlation between RT CIT-effect and BAC. The more intoxicated participants were, the larger the RT difference between their “no” responses to their own identity compared to their “no” responses to neutral stimuli. This result is in contrast to the idea that alcohol reduces attention and processing of the probes and strengthens the idea that the time-consuming process of inhibiting the truthful response contributes to the RT-CIT effect. Of course, the influence of alcohol on response inhibition is only one explanation for the observed pattern. Other theoretical explanations are possible, with an example being that instead of purely reducing attention, alcohol may rather cause a shift towards a less focused, yet more diffuse and distributed attention, which may in the CIT result in decreased performance on targets, but at the same time facilitate processing of probe items^26^. Further analyses on the performance on target trials, that primarily served to investigate a potential more general effect of alcohol on working memory capacity and action selection do, however, not support such an explanation. Those analyses revealed no significant correlations between alcohol consumption and the performance on target trials (using the performance on neutral trials as baseline). Note that on the basis of these results, we can of course not rule out that correlations may turn significant with larger power (especially with the low p-value in the correlation in the error rate). However, those results do point in the direction that such correlations and thereby general effects of alcohol on working memory capacity and action selection may not be as strong as the effects of alcohol on response inhibition (which is needed for a correct performance on probes and not on targets). Unfortunately, our field design did not allow for a random allocation of BACs to participants, and due to strict time constraints, we could not asses potentially confounding variables such as impulsivity or general drinking behavior. Importantly, impulsivity did not correlate with acute alcohol intoxication in a similar field design^13^ and accumulating evidence suggests that it does represent an inhibition component that is different from the type of motor inhibition that is hypothesized to underlie RT-CIT effects^27–30^. In contrast, habitual alcohol intoxication has been shown to correlate with acute alcohol intoxication in a similar field design^13^ and also with response inhibition capacities^31,32^. We therefore cannot exclude that habitual alcohol use may have contributed to (but most likely does not fully explain, see ref.^13^) the increase in the RT-CIT effect, yet this does not necessarily invalidate the conclusion that response inhibition processes are driving RT CIT-effects. It would be interesting for further research to investigate whether this conclusion also holds for other types of concealed information. In the current experiment, we used very salient and deeply encoded identity information. Although there are situations in which such identity information is the information that investigators want to reveal (e.g., in cases where the identity of a suspect is unknown or an examinee is suspected of hiding his/her actual identity), such cases do not represent the most typical scenario for CIT use. More frequent situations in the field concern settings where involvement in a crime should be determined by assessing knowledge of crime details in a CIT. Research investigating the effects of alcohol on attentional processes suggests that such effects may be mediated by effects of alcohol on working memory^33,34^. It would therefore be of theoretical as well as applied interest to investigate how acute alcohol intoxication would modulate attention to less-deeply encoded information (e.g., information that has been incidentally acquired during a [mock] crime). Under such circumstances, alcohol may have more pronounced negative effects on memory and recall performance, which may, in turn, decrease response costs for critical information.

Further research should also assess potential dissociations between different concealed information measures (e.g., RTs and autonomic response measures). Interestingly, whereas alcohol has been found to impair attention measured in behavioral tasks or attention-related event-related potentials, it has also been shown to increase autonomic indices of the orienting response^35^. This would be especially interesting considering that recent research suggests a stronger influence of response inhibition on behavioral and neural measures of concealed information compared to autonomic measures^16^.

The current study provides first evidence for the impact of alcohol on the detection of concealed information from behavioral responses. This is not only interesting from the above mentioned theoretical perspective, but may also be relevant in applied contexts in which suspects or eye-witnesses may incidentally be intoxicated during first interrogations or when potential suspects may secretly use alcohol in the hope to “defeat” the test. If such alcohol use remains undetected or when CIT examinations cannot be postponed (e.g., in cases of terroristic threats or abductions), the current results indicate that the test is still valid and yields even larger RT CIT-effects. It should be explicitly noted though that we do not suggest to conclude from our results that alcohol should be given to examinees in order to increase CIT-effects. Aside from the fact that the effect of alcohol on innocent examinees is still unknown, ethical considerations definitely prevent such practice.

A few limitations of the current study should be mentioned. First, our results are in contrast with data from Bradley and Ainsworth (1984)^7^, who did not find effects of alcohol intoxication on autonomic responses in the CIT. Although these contrasting results may be explained by the considerably smaller sample size in the study of Bradley and Ainsworth (1984; *n* = 8 in the group that was intoxicated during the test) and by the aforementioned potential differences between behavioral and physiological measures, further research is necessary to explore those possibilities. Second, with *n* = 42, also the sample size of our study was restricted. Although there was a relatively high number of participants with zero BAC, the remaining BAC levels showed a sufficient variability across a relatively large range, indicating that the current sample was appropriate for estimating correlations with CIT responses. A replication of the study, preferably by another research group, is, however, indispensable to gain more reliable information in the existence and the size of the true effect. Third, as mentioned above, our field design did not allow for a random allocation of participants to the experimental condition (i.e., BACs). We hope our findings encourage future research in more controlled laboratory settings using random allocations of participants to either an alcohol or a placebo group, which could shed light on the question which of the highly entangled factors like acute alcohol intoxication, habitual alcohol use or impulsivity traits most strongly affects CIT performance.

Taken together, the current results demonstrate that robust CIT effects can be obtained even when testing conditions differ from typical laboratory settings. The positive correlation of RT-CIT effects with BAC furthermore suggests that response inhibition contributes to the RT-CIT effect.
